# Block copolymer microparticles comprising inverse bicontinuous phases prepared *via* polymerization-induced self-assembly†
†Electronic supplementary information (ESI) available: Experimental protocols, DMF GPC curves, SEM and TEM images, SAXS, BET raw data and summary table of the control experiments. See DOI: 10.1039/c9sc00303g


**DOI:** 10.1039/c9sc00303g

**Published:** 2019-03-11

**Authors:** Pengcheng Yang, Yin Ning, Thomas J. Neal, Elizabeth R. Jones, Bryony R. Parker, Steven P. Armes

**Affiliations:** a Department of Chemistry , University of Sheffield , Brook Hill , Sheffield , South Yorkshire S3 7HF , UK . Email: p.yang.simon@gmail.com ; Email: Y.Ning@sheffield.ac.uk ; Email: s.p.armes@sheffield.ac.uk

## Abstract

Traditionally, post-polymerization processing routes have been used to obtain a wide range of block copolymer morphologies. However, this self-assembly approach is normally performed at rather low copolymer concentration, which precludes many potential applications. Herein, we report a facile method for the preparation of block copolymer particles exhibiting complex internal morphology *via* polymerization-induced self-assembly (PISA). More specifically, a series of diblock copolymers were synthesized by reversible addition–fragmentation chain transfer (RAFT) alternating copolymerization of styrene (St) with *N*-phenylmaleimide (NMI) using a poly(*N*,*N*-dimethylacrylamide) (PDMAC) stabilizer as a soluble precursor. Conducting such PISA syntheses in a 50 : 50 w/w ethanol/methyl ethyl ketone (MEK) mixture leads directly to the formation of *micrometer-sized* PDMAC-P(St-*alt*-NMI) diblock copolymer particles at 20% w/w solids. Adjusting the degree of polymerization (DP) of the core-forming P(St-*alt*-NMI) block to target highly asymmetric copolymer compositions provides convenient access to an inverse bicontinuous phase. TEM studies of intermediate structures provide useful insights regarding the mechanism of formation of this phase. SEM studies indicate that the final copolymer particles comprise perforated surface layers and possess nanostructured interiors. In addition, control experiments using 1,4-dioxane suggest that the high chain mobility conferred by the MEK co-solvent is essential for the formation of such inverse bicontinuous structures. One-pot PISA formulations are reproducible and involve only cheap, commercially available starting materials, so they should be readily amenable to scale-up. This augurs well for the potential use of such nanostructured micrometer-sized particles as new organic opacifiers for paints and coatings.

## Abstract 2

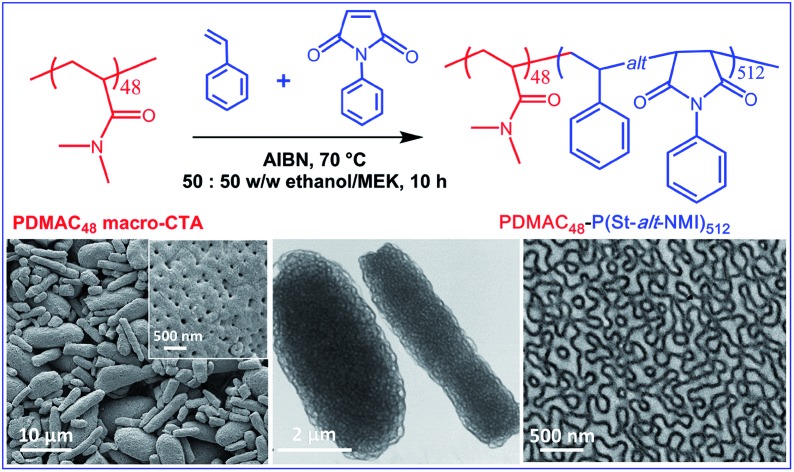
Scalable preparation of micrometer-sized diblock copolymer particles exhibiting complex internal structure is achieved by RAFT-mediated polymerization-induced self-assembly (PISA).

## Introduction

It is well-known that AB diblock copolymers undergo spontaneous self-assembly to form ordered nanostructures both in the bulk^1^ and also in solution.^2^ Microphase separation is primarily driven by an unfavorable enthalpic interaction between the two blocks, which outweighs the relatively small entropy term. Block copolymer self-assembly in the bulk is usually conducted by annealing the initial structure above the glass transition temperature to achieve thermodynamic control and hence equilibrium morphologies. It is well-known that this strategy provides access to a wide range of morphologies, including 2D or 3D periodic structures with long-range order.^1^,^3^ In addition, traditional post-polymerization processing routes in dilute solution usually lead to the formation of spherical micelles,^4^,^5^ cylindrical micelles,^6^,^7^ lamellae^8^ or vesicles.^9^,^10^ Considerable attention has been devoted to the direct formation of structurally complex block copolymer nanoparticles using phase inversion.^11^ For example, linear,^12^–^15^ comb-like^16^–^18^ or dendritic^19^,^20^ amphiphilic diblock copolymers can self-assemble to form inverse bicontinuous phases in dilute solution. These aggregates are structurally similar to lipid cubosomes, and offer potential applications for templating, separation, catalysis and controlled release systems *etc.*^21^–^23^

According to the packing parameter concept introduced by Israelachvili and co-workers for small molecule surfactants,^24^ inverted phases can be obtained when the packing parameter, *P* exceeds unity. The packing parameter is defined by the equation *P* = *v*/*a*_0_*l*_c_, where *a*_0_ is the area of the hydrophilic head-group and *v* and *l*_c_ are the volume and length of the hydrophobic component, respectively. Originally introduced to account for the diverse range of surfactant structures that can be formed in aqueous solution, this purely geometric concept was subsequently extended to include block copolymer self-assembly.^25^,^26^ Thus, targeting a highly asymmetric amphiphilic diblock copolymer (*e.g.* a very long hydrophobic block coupled with a relatively short hydrophilic block) provides a *P* value greater than unity. Moreover, the copolymer architecture also has a profound influence on the packing parameter. For example, block copolymers possessing a branched hydrophobic block show a strong tendency to form inverse morphologies in dilute solution.^27^ Recent advances in synthetic polymer chemistry such as controlled radical polymerization^28^ and click chemistry^29^,^30^ have enabled precise control over copolymer composition and architecture. Nevertheless, the traditional approach used to prepare inverse copolymer morphologies remains both time-consuming and inefficient. Typically, extremely long annealing times are required to attain thermodynamically stable inverse structures. Moreover, the relatively high molecular weight of the hydrophobic block means that the required highly asymmetric block copolymer chains often become kinetically-trapped during the processing step, which can prevent convenient access to well-defined inverse morphologies.^31^ In addition, copolymer concentrations are invariably rather low (typically below 1.0% w/w), which unfortunately precludes many, if not all, potential commercial applications.

Over the past decade, polymerization-induced self-assembly (PISA) has provided a versatile new approach for the efficient preparation of block copolymer nano-objects in the form of concentrated colloidal dispersions.^32^–^38^ PISA typically involves synthesizing a soluble homopolymer *via* RAFT solution polymerization^39^–^41^ followed by chain extension of this precursor with a second block that becomes insoluble when it exceeds a certain critical length. This leads to the *in situ* formation of sterically-stabilized diblock copolymer nanoparticles. Classical morphologies (*e.g.* spheres, worms, lamellae or vesicles) similar to those obtained *via* traditional post-polymerization processing routes can be readily prepared *via* PISA at up to 50% w/w solids.^42^ Moreover, highly convenient “one-pot” protocols have been reported for some PISA formulations.^42^–^46^ However, as far as we are aware, there are only a few studies of the PISA synthesis of inverse morphologies such as hexagonally-packed hollow hoops (HHH)^47^ and porous nanospheres,^48^–^50^ which have been reported for polystyrene-based diblock copolymers. Unfortunately, such formulations typically suffer from relatively slow rates of polymerization, which inevitably result in substantially incomplete monomer conversions. For example, less than 15% styrene conversion was achieved after 48 h at 80 °C when preparing the HHH phase.^47^ This problem renders such PISA formulations unsuitable for commercial scale-up owing to the prohibitive cost of removing unreacted styrene monomer.

In the present study, we report the PISA synthesis of various inverse structures in the form of microparticles at 20% w/w solids using a much more efficient PISA formulation. Previously, we reported the preparation of conventional diblock copolymer nanoparticles *via* RAFT dispersion alternating copolymerization of styrene (St) with *N*-phenylmaleimide (NMI) utilizing a 50 : 50 w/w ethanol/MEK mixture and a non-ionic poly(*N*,*N*′-dimethylacrylamide) (PDMAC) stabilizer.^51^ The resulting P(St-*alt*-NMI) core-forming block has a relatively high *T*_g_ (219 °C),^52^ which leads to the formation of oligolamellar vesicles (OLV) during PISA, as well as the more typical sphere and worm phases. The MEK co-solvent aids solubilization of the NMI monomer and solvates the core-forming block within the growing diblock copolymer micelles, thus enhancing the mobility of the growing P(St-*alt*-NMI) chains. Taking advantage of such high chain mobility, herein we target more asymmetric diblock copolymer compositions to investigate self-assembly beyond the bilayer phase. Three inverse bicontinuous phases were formed by such PDMAC-P(St-*alt*-NMI) chains, with each morphology depending on the relative volume fraction of the P(St-*alt*-NMI) core-forming block. Moreover, intermediate species observed by TEM provide useful insights regarding the likely mechanism for the evolution in copolymer morphology during these PISA syntheses. Furthermore, TEM studies of selected ultramicrotomed microparticles reveal complex internal structures owing to their inverse bicontinuous phases. Finally, we demonstrate that it is possible to fabricate such nanostructured microparticles *via* PISA using a highly convenient “one-pot” synthetic protocol.

## Results and discussion

**Scheme 1:**
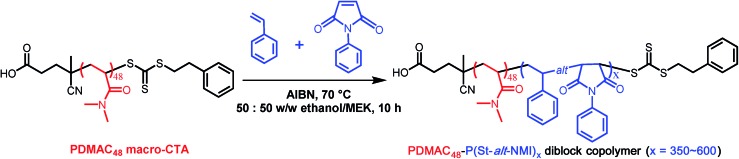
Reaction scheme for the RAFT alternating copolymerization of styrene (St) with *N*-phenylmaleimide (NMI) under dispersion polymerization conditions using a soluble poly(*N*,*N*-dimethylacrylamide) (PDMAC_48_) precursor in a 50 : 50 w/w ethanol/MEK solvent mixture at 70 °C.

**Fig. 1:**
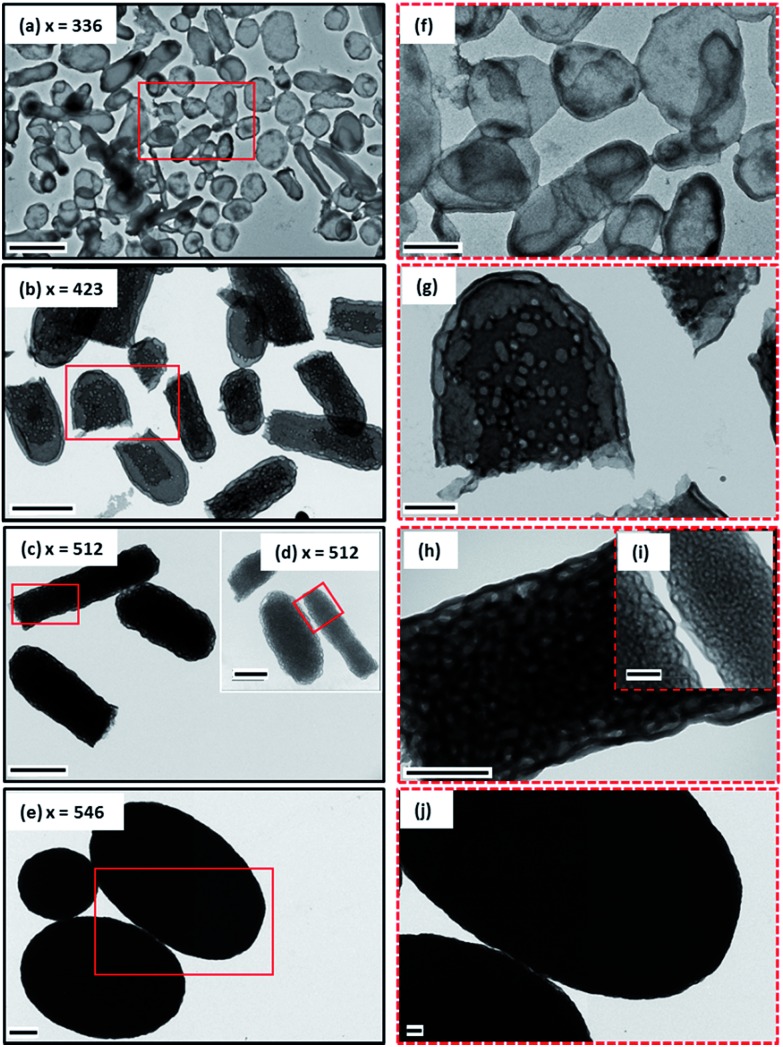
Representative TEM images (a–e) illustrating the evolution in copolymer morphology that occurs for a series of PDMAC_48_-P(St-*alt*-NMI)_*x*_ diblock copolymer microparticles prepared at 70 °C using a 50 : 50 w/w ethanol/MEK mixture *via* RAFT alternating dispersion copolymerization at 20% w/w solids. Higher magnification images (f–j) reveal the internal structure of these micrometer-sized particles. Scale bars correspond to 2 μm (a–e) and 0.50 μm (f–j).

The PDMAC stabilizer block DP was fixed at 48, and all RAFT dispersion alternating copolymerizations were conducted at 20% w/w solids in a 50 : 50 w/w ethanol/MEK mixture (see Scheme 1). These syntheses were much more efficient than the styrene-based methanolic formulations reported in the literature:^47^–^49^ more than 90% conversion was achieved in all cases within 10 h at 70 °C (see Table 1). DMF GPC studies indicated unimodal but relatively broad molecular weight distributions (see Fig. S1, ESI†). However, these *M*_w_/*M*_n_ values are comparable with those previously reported for the same PISA formulation,^51^ especially given that higher degrees of polymerization are targeted in the present study. The somewhat broader molecular weight distribution is most likely owing to relatively slow activation of the PDMAC_48_ stabilizer block rather than loss of RAFT end-groups, as previously suggested.^51^,^53^ GPC studies indicate a high blocking efficiency for these diblock copolymers, suggesting minimal homopolymer contamination. In principle, polydisperse diblock copolymers may behave differently to near-monodisperse copolymers in terms of their microphase separation. However, it is well-known that broad copolymer molecular weight distributions do not prevent (and may well aid) block copolymer self-assembly in the solid state.^54^–^56^ According to our earlier study, targeting a PDMAC_48_-P(St-*alt*-NMI)_350_ composition produced *micrometer-sized* ellipsoidal particles (see Fig. 1a).^51^ Small angle X-ray scattering (SAXS) analysis indicated that these relatively large particles comprised oligolamellar vesicles (OLV), comprising two or three stacked lamellae on average.^51^ This finding is consistent with the higher contrast (darker) regions observed by TEM in the present study (see Fig. 1f). Interestingly, targeting a P(St-*alt*-NMI) DP of 450 overshoots the OLV phase, generating polydisperse ellipsoidal particles with internal morphologies (see Fig. 1b). These ellipsoidal particles are relatively large: laser diffraction studies indicated a ‘sphere-equivalent’ volume-average diameter of around 9 μm, compared to just 2.6 μm diameter for the previously-reported OLV nano-objects.^51^ Internal structure can be clearly observed at higher TEM magnification (see Fig. 1g), with evidence for spherical (or elongated spherical) domains ranging from *ca.* 30 to 290 nm diameter. As far as we are aware, this morphology has not been reported previously for PISA syntheses, so we suggest the term perforated ellipsoidal lamellae (PEL). Further increasing the target core-forming DP to 550 for the core-forming block led to the formation of even larger ellipsoidal particles with a ‘sphere-equivalent’ diameter of around 12 μm (see Fig. 1c and d). Their internal morphology is difficult to characterize by TEM using an accelerating voltage of 80 kV (see Fig. 1h). However, using a 200 kV TEM instrument increases the penetration depth of the electron beam significantly, revealing a complex bicontinuous internal morphology (see Fig. 1d and i). Finally, ellipsoids with a ‘sphere-equivalent’ diameter of 14 μm were obtained when targeting a core-forming block DP of 600. However, TEM studies could not reveal the internal morphology of these microparticles (see Fig. 1e, j and S2, ESI†). Each of these dispersions formed turbid pastes at 20% w/w solids.

**Table 1:** Summary of the synthesis conditions and characterization data obtained for a series of PDMAC_48_-P(St-*alt*-NMI)_*x*_ diblock copolymer microparticles prepared *via* RAFT dispersion alternating copolymerization of styrene with *N*-phenylmaleimide at 70 °C using AIBN initiator in an 50 : 50 w/w ethanol/MEK mixture at 20% w/w solids^*a*^

Entry no.	Target DP for core-forming P(St-*alt*-NMI) block	Overall comonomer conversion^*b*^ (%)	Actual DP for core-forming P(St-*alt*-NMI) block^*c*^	DMF GPC		Laser diffraction volume-average diameter (μm)	BET surface area (m^2^ g^–1^)
				*M*_n_	*M*_w_/*M*_n_		
1	350	96	336	52 100	1.65	2.60 ± 3.67	31
2	400	97	388	55 300	1.64	4.32 ± 3.55	n.d.
3	425	95	404	58 100	1.65	7.89 ± 8.16	n.d.
4	450	94	423	61 200	1.66	9.00 ± 9.03	42
5	500	93	465	64 700	1.67	10.35 ± 11.20	n.d.
6	550	93	512	69 800	1.67	12.38 ± 12.73	53
7	600	91	546	72 300	1.69	14.31 ± 11.75	49
8^*d*^	650	96	624	85 800	1.62	4.92 ± 3.87	n.d.

^*a*^Conditions: [St]/[NMI] comonomer feed molar ratio = 1.0; [macro-CTA]/[AIBN] molar ratio = 10.

^*b*^Determined by ^1^H NMR spectroscopy in d_6_-DMSO.

^*c*^Actual DP of P(St-*alt*-NMI) = target DP of P(St-*alt*-NMI) × overall comonomer conversion.

^*d*^“One-pot” PISA synthesis conducted using a PDMAC_53_ macro-CTA.

**Fig. 2:**
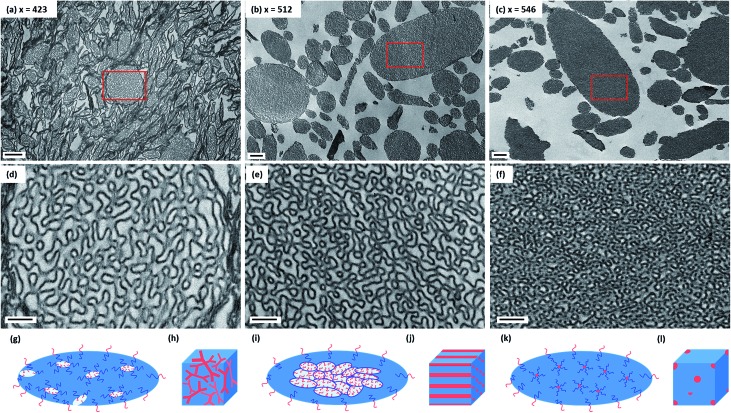
Cross-section TEM images (a–c) and corresponding higher magnification images (d–f) for ultramicrotomed PDMAC_48_-P(St-*alt*-NMI)_*x*_ microparticles embedded in epoxy resin reveal their complex internal morphologies. Schematic cartoons of various inverse structures: (g) perforated ellipsoidal lamellae, PEL; (i) bicontinuous ellipsoids, BE; (k) large compound micelles, LCM. Corresponding inverse morphologies for AB diblock copolymers in the bulk are: (h) bicontinuous gyroid; (j) hexagonally-packed cylinders; (l) body-centered cubic spheres. Scale bars correspond to 2 μm (a–c) and 0.5 μm (d–f).

Given the difficulty in analyzing their internal structure, the larger microparticles were embedded in an epoxy resin and ultramicrotomed to produce thin cross-sections for TEM studies. According to Fig. 2 (see also Fig. S3, ESI†), each ellipsoid is hollow: the darker regions correspond to the P(St-*alt*-NMI) copolymer, while the lighter regions represent internal voids. In general, increasing the DP of the P(St-*alt*-NMI) core-forming block produces smaller voids. This is in good agreement with the greater electron opacity observed for such particles in the initial TEM studies (see Fig. 1). The internal structure is illustrated at higher magnification in Fig. 2d–f. At first sight, the internal segregation observed for the PEL (see Fig. 2d) appears to be analogous to the well-known gyroid phase reported for block copolymers in the bulk^1^ (see Fig. 2h). This corresponds to an inverted mixed phase comprising worms and lamellae (Fig. 2d). Similarly, the bicontinuous ellipsoids (BE) particles (see Fig. 2e) seem to correspond to an inverse worm phase (see Fig. 2j), with PDMAC stabilizer chains forming the cores surrounded by P(St-*alt*-NMI) coronal blocks (see Fig. 2i). In this context, we note that Eisenberg's group were the first to describe the formation of inverted worms in dilute solution by self-assembly of highly asymmetric poly(acrylic acid)-poly(styrene) (PAA-PS)^57^ or poly(ethylene oxide)-poly(styrene) (PEO-PS)^58^,^59^ diblock copolymers. Such nano-objects exhibit a complex internal structure comprising hexagonally-packed hollow hoops or rods (HHH or HHR) distributed within a PS matrix. Moreover, Pan and co-workers recently reported the preparation of a HHH phase *via* the RAFT dispersion polymerization of styrene.^47^ In this latter case, the highly regular arrangement of the hollow hoops was verified by analysis of TEM cross-sections.^47^ On the other hand, it seems that PDMAC chains are just randomly packed within a P(St-*alt*-NMI) matrix for the BE microparticles obtained in the present study, with some spherical microdomains being observed (see Fig. 2e). The PDMAC_48_-P(St-*alt*-NMI)_546_ copolymer chains form large compound micelles (LCM)^60^ comprising isolated islands of the PDMAC stabilizer block located within a continuous P(St-*alt*-NMI) phase (see Fig. 2f). This internal structure resembles the inverted spheres (see Fig. 2l) that are formed by diblock copolymers in the bulk, where the relatively short hydrophilic PDMAC blocks are located within the cores and are surrounded by relatively long hydrophobic P(St-*alt*-NMI) coronal blocks (see Fig. 2k).

**Fig. 3:**
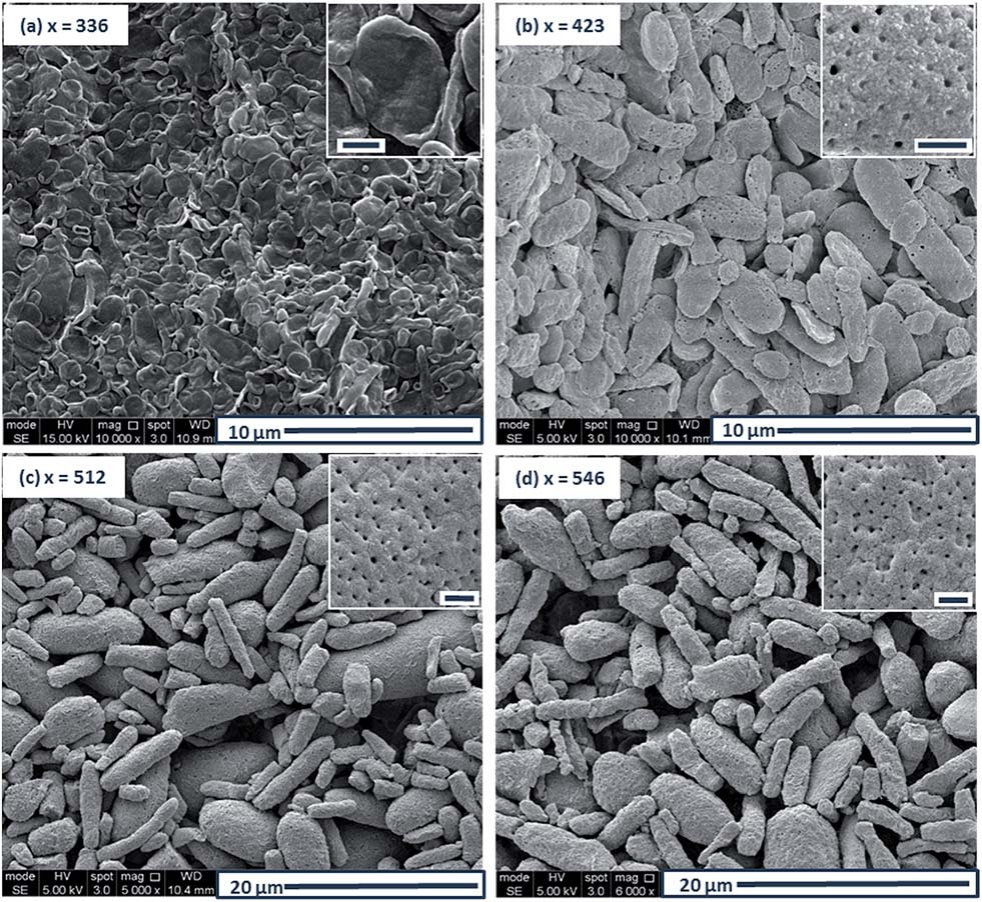
Representative SEM images illustrating the evolution in copolymer morphology for a series of PDMAC_48_-P(St-*alt*-NMI)_*x*_ diblock copolymers prepared *via* PISA at 70 °C using a 50 : 50 w/w ethanol/MEK mixture: (a) oligolamellar vesicles, OLV; (b) perforated ellipsoidal lamellae, PEL; (c) bicontinuous ellipsoids, BE; (d) large compound micelles, LCM. Insets show high magnification images of the surface morphology, with each scale bar corresponding to 500 nm.

**Fig. 4:**
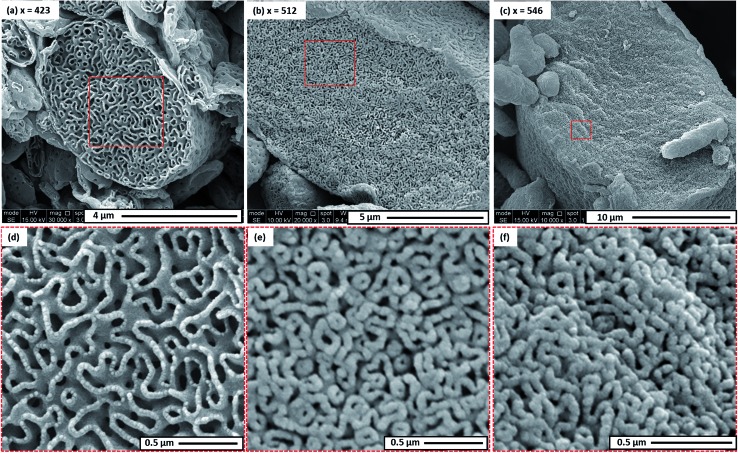
(a–c) High resolution SEM images obtained for selected randomly-fractured PDMAC_48_-P(St-*alt*-NMI)*_x_* microparticles (where *x* = 423, 512 or 546, respectively) revealing their bicontinuous internal structure. (d–f) High magnification images obtained for the internal morphologies shown in (a–c), respectively.

The morphology of this series of PDMAC_48_-P(St-*alt*-NMI)_*x*_ microparticles was also studied by SEM (see Fig. 3 and S4–S7, ESI†). Low-magnification images clearly indicate the progressive growth in mean particle size and evolution in copolymer morphology that occur on systematically increasing the target DP of the P(St-*alt*-NMI) core-forming block (see Fig. 3). High-resolution SEM images showed that all inverse phase microparticles are actually enclosed within a perforated surface layer (see Fig. 3b–d, inset). Based on these SEM images, the average surface pore dimensions are estimated to be approximately 100 ± 23 nm, 72 ± 17 nm and 71 ± 12 nm for PEL, BE and LCM, respectively. Conversely, no surface pores were discernible for OLV particles (Fig. 3a, inset). This observation suggests that initial phase separation occurs during the OLV-to-PEL transition (see later). The internal structure of selected microparticles was also studied by SEM by sectioning the microparticles using a razor blade (see Fig. 4). Their original morphology remains intact under the ultrahigh vacuum conditions required for SEM analysis. This is primarily owing to the relatively high *T*_g_ (219 °C) of the hydrophobic P(St-*alt*-NMI) block. More interestingly, examination of cross-sections of these fractured microparticles confirmed that their internal structure is bicontinuous and bears a superficial resemblance to a triply periodic minimal surface (see Fig. 4). A *minimal surface* has its local area minimized, *i.e.*, every point has zero mean curvature.^61^ Triply periodic minimal surfaces are periodic in all three coordinate directions. At first sight, the internal structure of PEL (see Fig. 4a) appears to be a Schoen^62^ gyroid surface. Increasing the DP of the P(St-*alt*-NMI) core-forming block to 512 (see Fig. 4b) or 546 (see Fig. 4c) results in an apparent transition from a Schoen gyroid surface to a Schwartz^63^ P surface. According to Thomas *et al.*,^64^ a high interfacial energy between the two blocks leads to strong segregation during diblock copolymer self-assembly. As microphase separation occurs, an area-minimizing surface is adopted in order to lower the total interfacial energy. Such minimal surfaces best satisfy this geometric constraint and have been observed for block copolymers both in melts^65^–^67^ and also in solution.^13^,^19^,^68^,^69^

To gain a better understanding of the internal structure of these microparticles, SAXS was used to characterize three copolymer samples (PEL, BE and LCM), both in their powder form and also as 1.0% w/w dispersions in ethanol. A characteristic structural peak at *q* ≈ 0.02 Å was observed for the dispersions in each case (see Fig. S8, ESI†). This *q* value corresponds to a length scale of approximately 31 nm, which is attributed to the thickness of the continuous phase. As shown in Fig. 2d–f, TEM studies of cross-sectioned microparticles indicate that the thickness of the continuous copolymer phase is ∼46 nm for all three copolymers, regardless of the P(St-*alt*-NMI) block DP. However, TEM overestimates this characteristic length scale owing to an intrinsic artefact of the sample preparation.^70^ The SAXS structure peaks are slightly shifted to higher *q* for the corresponding dried powders owing to the absence of solvation. In addition, another strong peak was observed at *q* = 0.0051 Å^–1^ (corresponding to 123 nm) for PEL. This feature was shifted to *q* = 0.0063 Å^–1^ (corresponding to 100 nm) for BE but becomes much less prominent for LCM (see Fig. S8, ESI†). This latter peak is characteristic of the internal porosity within these microparticles, with the mean pore size being in good agreement with TEM images (see Fig. 2d–f). Moreover, its breadth suggests a relatively broad pore size distribution. In summary, these SAXS studies confirm that these microparticles possess internal porosity but do *not* support the presence of triply periodic minimal surfaces.

**Fig. 5:**
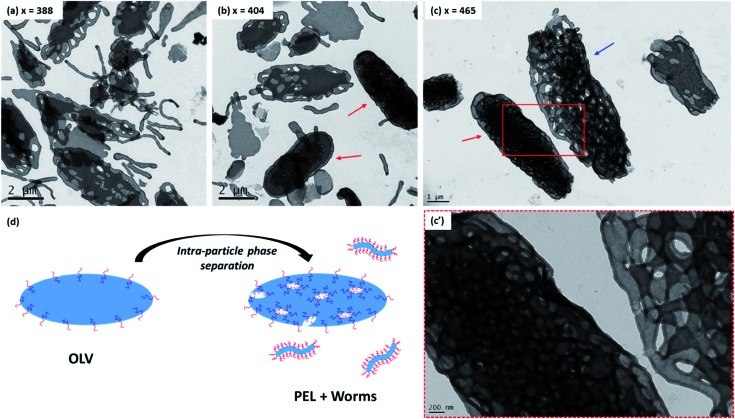
Representative TEM images obtained for intermediate morphologies formed during the PISA synthesis of PDMAC_48_-P(St-*alt*-NMI)_*x*_ microparticles at 70 °C in a 50 : 50 w/w ethanol/MEK mixture at 20% w/w solids. (a, b) The oligolamellar vesicle (OLV) to perforated ellipsoidal lamellae (PEL) transition (entries 2 & 3, Table 1); arrows indicate PEL particles, possibly formed *via* fusion/stacking of hollow bilayers. (c) The PEL to bicontinuous ellipsoid (BE) transition (entry 5, Table 1); red arrow indicates a relatively compact BE, possibly formed *via* intraparticle rearrangement from looser, more open structures such as that indicated by the blue arrow. (d) Schematic mechanism proposed for the initial phase separation, which involves expulsion of worms from the initial OLVs.

For the current PISA formulation, the evolution in morphology from bilayers to various inverted morphologies can be readily rationalized in terms of molecular curvature,^26^ which is in turn determined by the relative volume fractions of each block. Initially, the diblock copolymer morphology is OLV. This means that the relative volume fractions of the PDMAC block and the P(St-*alt*-NMI) block are approximately equal, hence low curvature interfaces are formed. Increasing the DP of P(St-*alt*-NMI) block increases its effective volume fraction, leading to an increase in copolymer curvature. Like the well-established morphology transitions observed for normal phases,^71^ this higher copolymer curvature drives a change in morphology from bilayers to inverted cylindrical micelles (*i.e.* inverted worms) and inverted spheres. The observation of trapped intermediates (see Fig. 5) during the OLV to PEL to BE transitions suggest that the mechanism for formation of these latter morphologies involves three steps: (i) initial OLV phase separation (see Fig. 5a), which results in the formation of worm-like particles and hollow bilayer structures (see Fig. 5d); (ii) fusion/stacking of hollow bilayers to generate large aggregates (see arrows in Fig. 5b) – both TEM and SEM studies indicate a significant increase in particle size during the OLV to PEL transition (see Fig. 3); (iii) rearrangement to form more ordered internal structures (see arrow in Fig. 5c). This mechanism is similar to that proposed for the formation of HHH,^47^,^57^ but these earlier studies did not involve intra-particle phase separation in the initial stages. In addition, unlike HHH or porous nanospheres, which were prepared from spherical vesicle precursors, the current inverse structures are generated from self-assembly of micrometer-sized ellipsoidal lamellae (OLV). This explains why (i) the inverse structures obtained in the present study possess an ellipsoidal morphology and (ii) the resulting particles predominantly lie in the μm (rather than nm) size range.

Control experiments conducted using a 50 : 50 w/w ethanol/1,4-dioxane mixture under otherwise identical conditions (see Table S1, ESI†) yielded only conventional copolymer morphologies, *e.g.* spheres, worms and worm clusters (see Fig. S9a–c, ESI†). Increasing the target core-forming block DP did not afford inverted morphologies, but instead produced only ill-defined, colloidally unstable aggregates (see Fig. S9d, ESI†). As discussed in our previous study,^51^ MEK is a significantly better solvent for the structure-directing P(St-*alt*-NMI) block than 1,4-dioxane. Therefore, these results suggest that the high chain mobility conferred by the former co-solvent is essential to access inverted morphologies in such PISA formulations. This is consistent with various PISA syntheses involving polystyrene-based diblock copolymers reported in the literature.^47^–^49^ As styrene is a good solvent for polystyrene, the styrene-rich domains increase the chain mobility and facilitate the evolution in morphology towards inverted phases. In the present study, BET studies conducted using N_2_ gas as an adsorbate at 77 K indicate that these inverted structures possess significantly higher specific surface areas (42–53 m^2^ g^–1^) than that of the OLVs (31 m^2^ g^–1^), despite the larger size of the former particles (see Table 1). The higher specific surface areas observed for these porous microparticles indicate a reduction in internal void volume, which is consistent with electron microscopy observations (see Fig. 2 and 4). In addition, this relatively high surface area suggests that the nitrogen probe molecules used in the BET measurements can diffuse through surface pores in the perforated outer shell and hence access the internal bicontinuous network. The specific surface areas determined for these microparticles are approximately a factor of two lower than those reported by Kim and co-workers for similar diblock copolymer microparticles prepared *via* post-polymerization processing.^19^

**Fig. 6:**
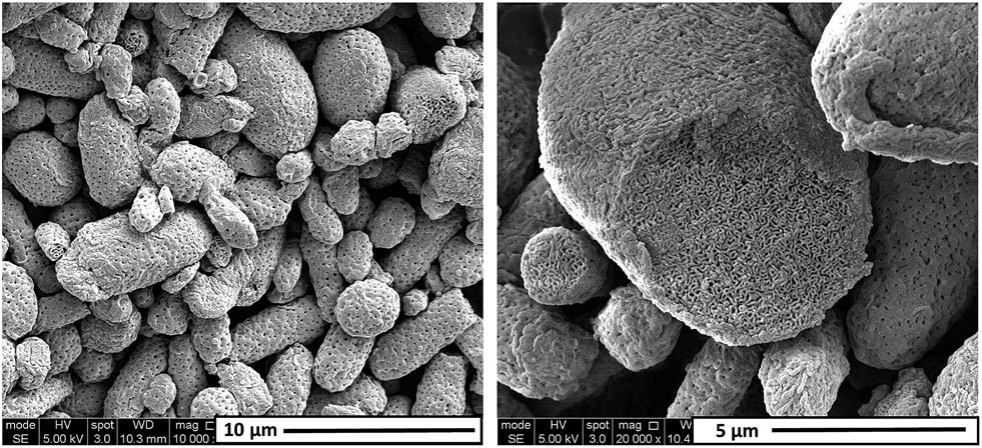
Representative SEM images recorded for PDMAC_53_-P(St-*alt*-NMI)_624_ microparticles prepared in a 50 : 50 w/w ethanol/MEK mixture at 20% w/w solids *via* a “one-pot” PISA protocol.

In principle, the ability to conveniently prepare micrometer-sized particles exhibiting an inverse bicontinuous morphology *via* PISA is an important advantage for potential industrial scale-up. Therefore, we investigated the feasibility of a “one-pot” synthesis of such particles, targeting a diblock composition of PDMAC_60_-P(St-*alt*-NMI)_650_ (entry 8, Table 1). More specifically, DMAC (target DP = 60; 40% w/w solids) was first polymerized to high conversion *via* RAFT solution polymerization at 70 °C in pure ethanol using 4-cyano-4-(2-phenylethanesulfanylthiocarbonyl)sulfanylpentanoic acid (PETTC) RAFT agent and AIBN initiator. After 2.5 h, this DMAC homopolymerization had attained 89% conversion. At this point, an equimolar mixture of styrene and NMI monomer in an MEK-rich ethanol/MEK mixture was added to the reaction vessel under nitrogen. For this one-pot protocol, the final composition of ethanol/MEK mixture was 50 : 50 w/w and the target copolymer concentration was 20% w/w solids. The alternating copolymerization was allowed to proceed for a further 10 h under RAFT dispersion polymerization conditions at 70 °C. Laser diffraction studies indicated that the final PDMAC_60_-P(St-*alt*-NMI)_650_ microparticles obtained from this “one-pot” synthesis protocol were somewhat smaller (*ca.* 4.92 μm) than the PDMAC_48_-stabilized microparticles described above. However, SEM studies confirmed that the former microparticles had a similar perforated surface layer (see Fig. 6, left) and, more importantly, exhibited a bicontinuous internal morphology (see Fig. 6, right). Moreover, ^1^H NMR studies indicated an overall comonomer conversion of 96% for this one-pot PISA protocol. This preliminary finding augurs well for the convenient synthesis of micrometer-sized particles with complex internal structures on a larger scale. In principle, these highly porous microparticles could be used as organic opacifiers for paint formulations. In this context, more intense light scattering could be achieved by optimizing the mean size of the internal voids so that they are approximately half the wavelength of visible light (*e.g.* for efficient scattering at a wavelength of 500–600 nm, the void dimensions should be 250–300 nm, which is somewhat larger than the voids currently achieved). A further requirement for this particular application is that the diblock copolymer microparticles must remain intact during solvent evaporation as the wet paint dries. This should be feasible for this particular PISA formulation given the relatively high *T*_g_ of the structure-directing P(St-*alt*-NMI) chains.

## Conclusions

In summary, polymerization-induced self-assembly has been exploited to prepare a range of PDMAC_48_-P(St-*alt*-NMI)_*x*_ diblock copolymer particles *via* RAFT dispersion alternating copolymerization of styrene with *N*-phenylmaleimide using a 50 : 50 w/w ethanol/MEK binary solvent mixture. TEM analysis confirmed that the resulting micrometer-sized particles possess an ellipsoidal morphology and complex internal nanostructures. For a fixed PDMAC stabilizer block DP, increasing the DP of the P(St-*alt*-NMI) block leads to a gradual evolution in morphology, from inverse gyroids (PEL) to inverse worms (BE) and finally to inverse spheres (LCM). SEM studies indicate that these inverse structures have highly porous surfaces with bicontinuous internal networks. The formation of such structures is most likely driven by minimization of the total interfacial energy. The mechanism for the formation of these inverse morphologies appears to be similar to that proposed by Eisenberg and co-workers, who obtained a HHH morphology *via* traditional post-polymerization processing of diblock copolymers in dilute solution.^47^,^57^ Control experiments utilizing 1,4-dioxane instead of MEK suggest that sufficiently high chain mobility is essential for achieving such inverse phases, otherwise only kinetically-trapped morphologies are obtained when increasing the relative volume fraction of the structure-directing block. Such nanostructured micrometer-sized particles can be conveniently synthesized at high solids *via* a “one-pot” PISA protocol using cheap starting materials and relatively benign solvents, so they may offer some potential as new organic opacifiers. However, such an application would require the mean size of the internal voids to be further optimized in order to maximize the light scattering and hence hiding power. This refinement is beyond the scope of the current study.

## Conflicts of interest

The authors declare no competing financial interest.

## Supplementary Material

Supplementary information
