# Supplementary material for: Block copolymer microparticles comprising inverse bicontinuous phases prepared via polymerization-induced self-assembly
Source: Chem Sci. 2019 Mar 11;10(15):4200–8. doi: 10.1039/c9sc00303g (PMC6460954; doi:10.1039/c9sc00303g)
Supplement: Supplementary file 1 [file SC-010-C9SC00303G-s001.pdf]

Electronic Supplementary Material (ESI) for Chemical Science.
This journal is © The Royal Society of Chemistry 2019

Supporting Information for:

Block copolymer microparticles comprising inverse bicontinuous phases prepared via polymerization-induced self-assembly

Pengcheng Yang,*[a†] Yin Ning,*[a] Thomas J. Neal,[a] Elizabeth R. Jones,[a] Bryony R. Parker,[a] and Steven P. Armes*[a]

[a] Department of Chemistry, University of Sheffield, Brook Hill, Sheffield, South Yorkshire, S3 7HF, UK.

E-mail: p.yang.simon@gmail.com; Y.Ning@sheffield.ac.uk; s.p.armes@sheffield.ac.uk

[†] Current address: Allnex, Nieuwe Kannal 7N, 6709 PA, Wageningen, the Netherlands.

Experimental

Materials. All reagents were purchased from Sigma-Aldrich (UK) and were used as received, unless otherwise noted. 2,2'-Azobisisobutyronitrile (AIBN, Molekular, Germany) was used as an initiator. *N*-Phenylmaleimide (NMI, 98%) was recrystallized from cyclohexane. Styrene (St, 99%) and *N,N*-dimethylacrylamide (DMAC, 99%) were each purified via column chromatography to remove inhibitor using basic alumina as the stationary phase, and then stored at -20 °C prior to use. Absolute ethanol (maximum water content = 0.1%) was supplied by VWR international S.A.S (Fontenay-sous-Bois, France). All other solvents were of HPLC quality and were supplied by Fisher Scientific (Loughborough, UK). All deuterated solvents were obtained from Goss Scientific (Cambridge, UK) and used as received. 4-Cyano-4-(2-phenylethanesulfanylthiocarbonyl)sulfanylpentanoic acid (PETTC) RAFT agent was prepared as described in the literature.[1]

Synthesis of poly(*N,N*-dimethylacrylamide) (PDMAC) macro-CTA. In a 100 ml round-bottomed flask, PETTC (0.856 g; 2.52 mmol), AIBN (0.0414 g; 0.252 mmol; CTA/initiator molar ratio = 10), and *N,N*-dimethylacrylamide (DMAC; 15.0 g; 151 mmol; target DP = 60) were dissolved in 1,4-dioxane (45.0 g) to obtain a 25% w/w DMAC solution. The reaction mixture was degassed using a dry nitrogen purge for 50 min at 0 °C before the reaction flask was immersed in a preheated oil bath at 70 °C. After 2.5 h (81% DMAC conversion), the DMAC polymerization was quenched by cooling the reaction mixture to 20 °C with subsequent exposure of the reaction solution to air. 1,4-dioxane (30 ml) was then added to dilute the solution, and unreacted DMAC monomer was removed by precipitation into a ten-fold excess of diethyl ether. This purification protocol was repeated twice. A yellow solid was obtained

after drying under vacuum (10.3 g, 69% yield; M_n = 5,800; M_w/M_n = 1.10). ^{1}H NMR spectroscopy indicated a mean degree of polymerization of 48 for the purified PDMAC macro-CTA in d_4-methanol (calculated by comparing the integrated aromatic signals assigned to the RAFT chain-ends at 7.1-7.4 ppm with those assigned to the acrylamide backbone at 1.0-2.0 ppm).

RAFT dispersion alternating copolymerization of styrene with *N*-phenylmaleimide using a $PDMAC_{48}$ macro-CTA in a 50/50 w/w ethanol/MEK mixture. In a typical formulation targeting $PDMAC_{48}$-P(St-*alt*-NMI)$_{450}$ at 20% w/w solids, $PDMAC_{48}$ macro-CTA (45.3 mg; 8.89 µmol), AIBN (0.146 mg; 0.889 µmol; CTA/initiator molar ratio = 10), styrene (208 mg; 2.00 mmol) and *N*-phenylmaleimide (346 mg; 2.00 mmol) were dissolved in a 50/50 w/w ethanol/MEK mixture (2.34 g). This reaction mixture was sealed in a 10 ml Schlenk flask and purged with nitrogen for 15 min at 20 ^oC, then the flask was immersed in a preheated oil bath at 70 ^oC. The RAFT alternating copolymerization was allowed to proceed for 10 h to ensure at least 90% total monomer conversion and then quenched by exposure to air. Other diblock copolymer compositions were targeted by adjusting the relative amounts of $PDMAC_{48}$ macro-CTA and the styrene/*N*-phenylmaleimide comonomer mixture (using a fixed mass of $PDMAC_{48}$ macro-CTA). In a series of control experiments, the MEK co-solvent was replaced with the same mass of 1,4-dioxane (see Table S1 and Figure S9).

One-pot synthesis of $PDMAC_{60}$-P(St-*alt*-NMI)$_{650}$ diblock copolymer via RAFT dispersion alternating copolymerization. In a 50 ml round-bottom flask, PETTC (17.1 mg; 50.4 µmol), AIBN (0.410 mg; 2.52 µmol; CTA/initiator molar ratio = 10), and *N,N*-dimethylacrylamide (DMAC; 0.300 g; 3.03 mmol; target DP = 60) were dissolved in pure ethanol (0.477 g) to obtain a 40% w/w DMAC solution. This reaction mixture was degassed using a dry nitrogen purge for 30 min at 0 ^oC then the flask was immersed in a preheated oil bath at 70 ^oC. After 2.5 h, a small aliquot (~ 50 µl) was removed for ^{1}H NMR studies (89% DMAC conversion). Then a previously degassed solution of styrene (2.84 g; 16.4 mmol) and *N*-phenylmaleimide (1.71 g; 16.4 mmol) dissolved in a mixture of ethanol (9.25 g) and MEK (9.73 g) was added. The second-stage alternating copolymerization was allowed to proceed for a further 10 h at 70 ^oC.

Copolymer characterization

^{1}H NMR spectroscopy. All ^{1}H NMR spectra were either recorded in d_4-methanol or d_6-DMSO using a 400 MHz Bruker Avance-400 spectrometer. Typically 64 scans were averaged per spectrum.

Gel permeation chromatography (GPC). Analyses were performed using an Agilent 1260 Infinity set-up equipped with two PL gel 5 µm Mixed-C columns and one Phenogel 5 µm linear/mixed guard maintained at 60 ˚C. The DMF eluent contained 10 mM LiBr, and the flow rate was 1.0 mL min^{-1}. Calibration was achieved using a series of ten near-monodisperse poly(methyl methacrylate) (PMMA) standards with M_p values ranging from 625 g mol^{-1} to 618,000 g mol^{-1}.

Laser diffraction. Diblock copolymer microparticles were sized using a Malvern Mastersizer 2000 instrument equipped with a small volume Hydro 2000SM sample dispersion unit (ca. 50 mL), a He-Ne laser operating at 633 nm and a solid-state blue laser operating at 466 nm. The stirring rate was adjusted to 1000 rpm.

Transmission electron microscopy (TEM). Imaging was performed using either a FEI Tecnai Spirit instrument at 80 kV equipped with a Gatan 1k CCD camera or a Philips CM200 200 kV electron microscope. Copper/palladium TEM grids (Agar Scientific, UK) were surface-coated to yield a thin film of amorphous carbon. These grids were then plasma glow-discharged for 30 seconds to create a hydrophilic surface. A small droplet (11 µL) of a dilute copolymer dispersion in ethanol was placed onto the freshly-prepared grids for 60 seconds and then carefully blotted with filter paper to remove excess solution. To stain the microparticles, a 0.75 % w/v uranyl formate solution (9 µL) was placed onto the sample-loaded grid via micropipet for 20 seconds and then carefully blotted to remove excess stain. The grids were then dried using a vacuum hose. To observe their internal structure, selected microparticles were embedded in an epoxy matrix and cured at 60 °C for three days. Ultrathin sections of approximately 100 nm thickness were then prepared using a Leica EMUG6 ultramicrotome equipped with a diamond knife at ambient temperature. Finally, these ultrathin sections were placed on copper grids for TEM examination.

Scanning electron microscopy (SEM). The morphology of diblock copolymer microparticles was examined by SEM (FEI Inspect F instrument) operating at an accelerating voltage of 5 to 15 kV. Samples were carefully sliced using a razor blade before gold coating (15 mA, 4 min).

BET surface area analysis. Selected dried diblock copolymer microparticles were analyzed by BET nitrogen adsorption at 77 K using a Nova 1000e Quantachrome instrument. The specific surface area (A_s, m^2 g^{-1}) was determined using the five-point BET method over a relative pressure (P/P_0) range of

0.10–0.30. A_s values were calculated using the Brunauer-Emmett-Teller (BET) equation assuming a parking area of 16.2 Å^2 for the dinitrogen molecule.

Small angle X-ray scattering (SAXS). SAXS data were collected using a laboratory-based Xenocs Xeuss 2.0 instrument equipped with a Dectris Pilatus 1M detector and an Excillum liquid gallium MetalJet X-ray source (X-ray wavelength, λ = 1.34 Å). SAXS patterns were recorded for a scattering vector, *q,* ranging from 0.0014 Å^{-1} to 0.3 Å^{-1} [he length of the scattering vector, *q*, is given by $q = \frac{4\pi}{\lambda}\sin\theta$, where ϑ is half the scattering angle]. X-ray scattering data were reduced, normalized and background-subtracted using Irena SAS macros for Igor Pro.[2]

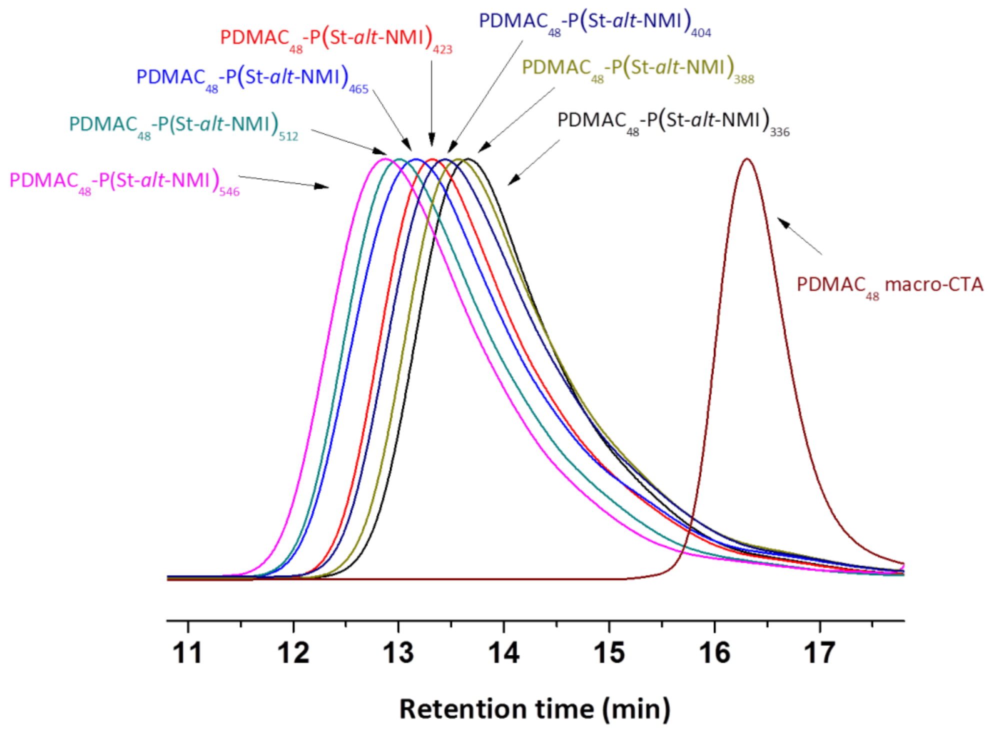

Figure S1. DMF GPC traces obtained for $PDMAC_{48}$ macro-CTA precursor and a series of $PDMAC_{48}$-P(St-*alt*-NMI)$_x$ diblock copolymers synthesized via RAFT dispersion alternating copolymerization of styrene and N-phenylmaleimide using a 50/50 w/w ethanol/MEK binary solvent mixture at 70 °C.

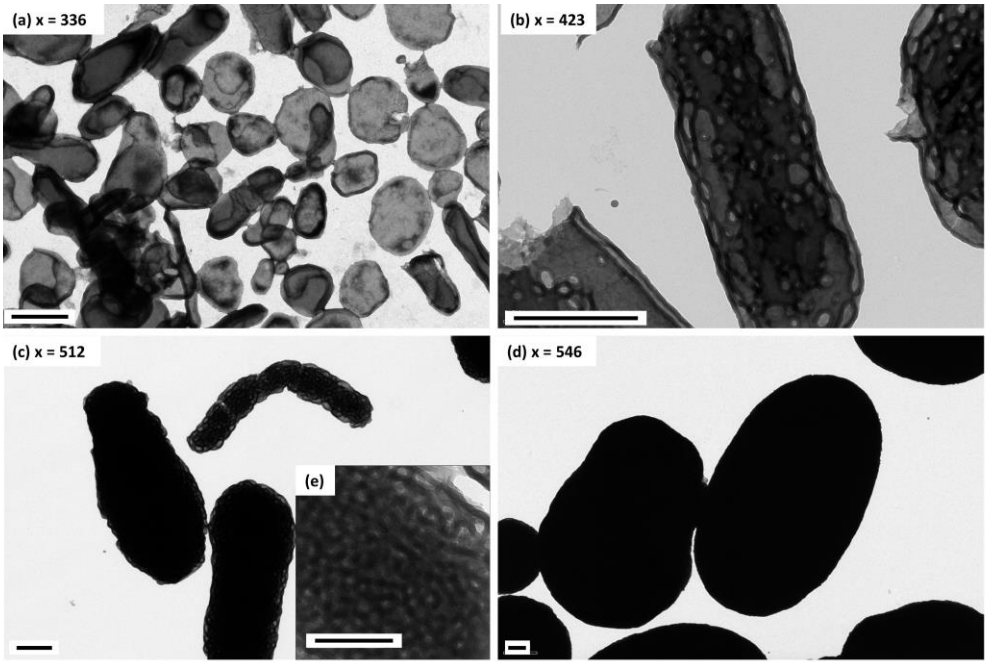

Figure S2. Four TEM images (and also a high magnification image of the internal structure, see inset e within c illustrating the evolution in copolymer morphology observed for a series of $PDMAC_{48}$-P(St-*alt*-NMI)$_x$ diblock copolymer microparticles prepared at 70 °C using a 50/50 w/w ethanol/MEK mixture via RAFT alternating dispersion copolymerization of styrene with *N*-phenylmaleimide at 20% w/w solids. Scale bars indicate either 1 µm (a-d) or 0.50 µm (e).

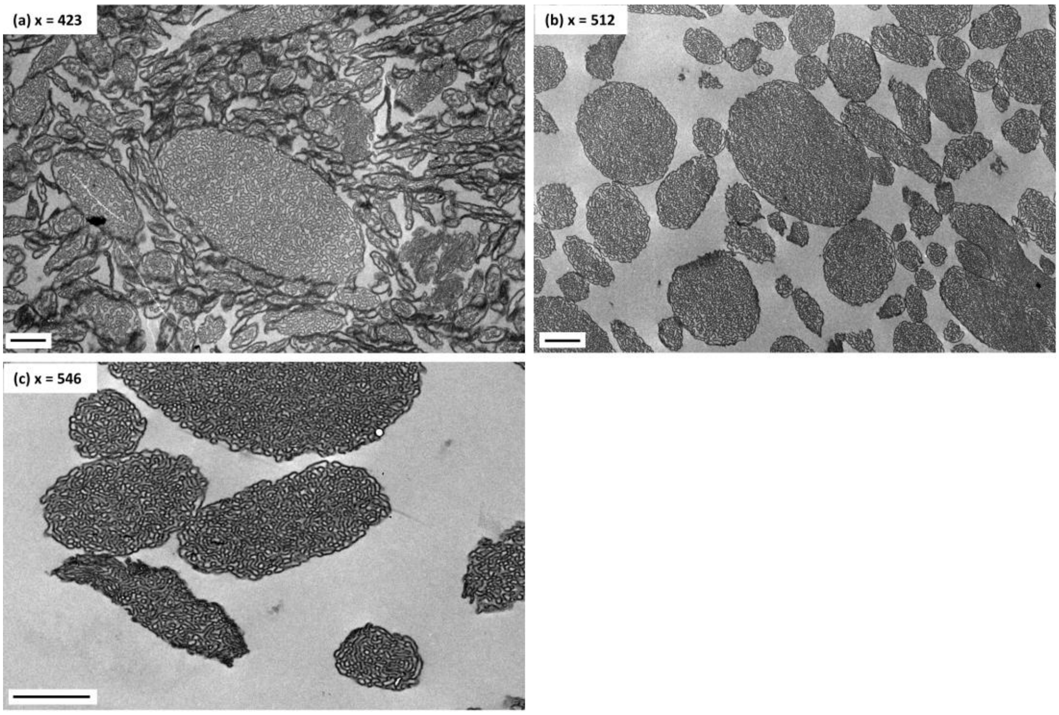

Figure S3. TEM images recorded after ultramicrotomy revealing the complex internal morphologies of three of the four PDMAC$_{48}$-P(St-*alt*-NMI)$_x$ diblock copolymer microparticles shown in Figure S2. Scale bars indicate 2 µm in each case.

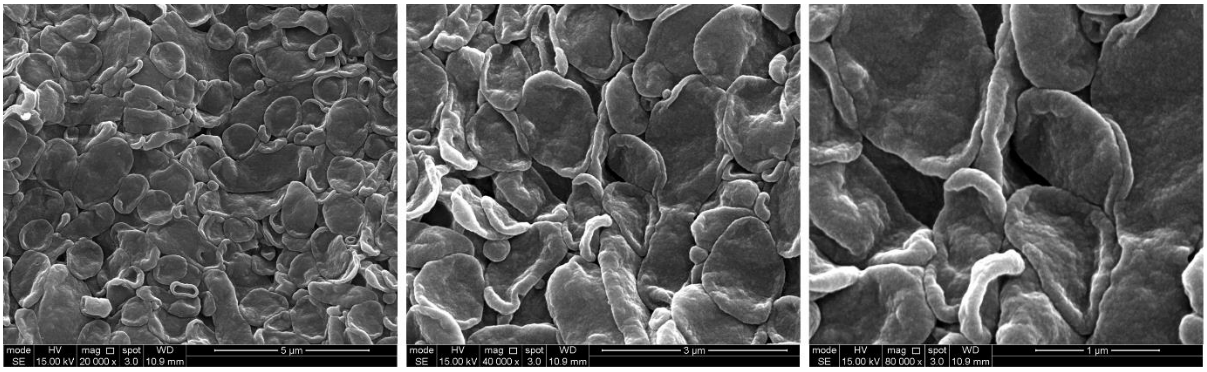

Figure S4. SEM images recorded for PDMAC$_{48}$-P(St-*alt*-NMI)$_{336}$ oligolamellar vesicles, OLV.

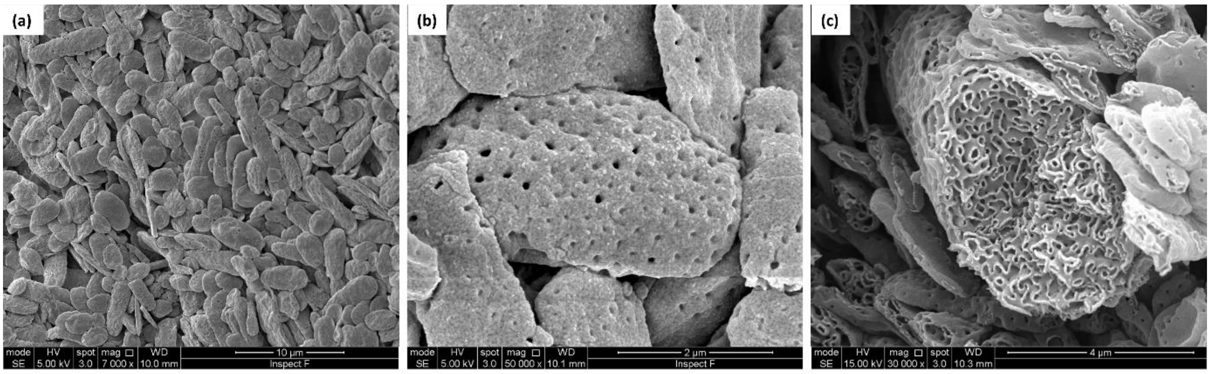

Figure S5. SEM images recorded for PDMAC$_{48}$-P(St-*alt*-NMI)$_{423}$ perforated ellipsoidal lamellae, PEL: (a) at low magnification; (b) at high magnification revealing their surface porosity; (c) randomly-fractured PELs showing the complex internal structure.

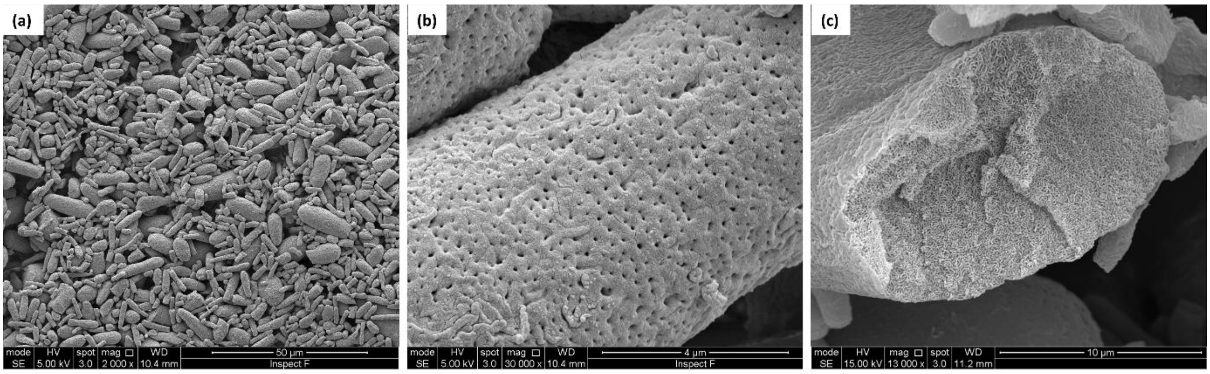

Figure S6. SEM images recorded for PDMAC$_{48}$-P(St-*alt*-NMI)$_{512}$ bicontinuous ellipsoids, BE: (a) at low magnification; (b) at high magnification revealing their surface porosity; (c) randomly-fractured BEs showing their complex internal structure.

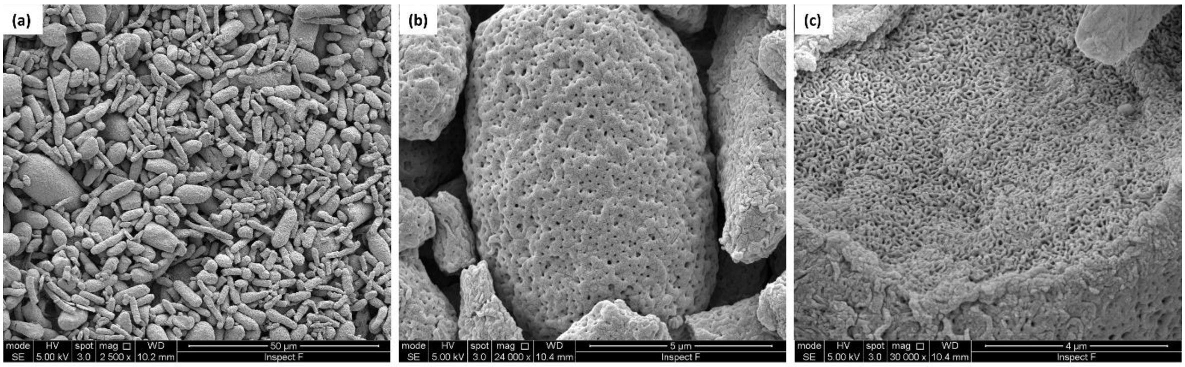

Figure S7. SEM images recorded for PDMAC$_{48}$-P(St-*alt*-NMI)$_{546}$ large compound micelles, LCM: (a) at low magnification; (b) at high magnification revealing their surface porosity; (c) randomly-fractured LCMs showing their complex internal structure.

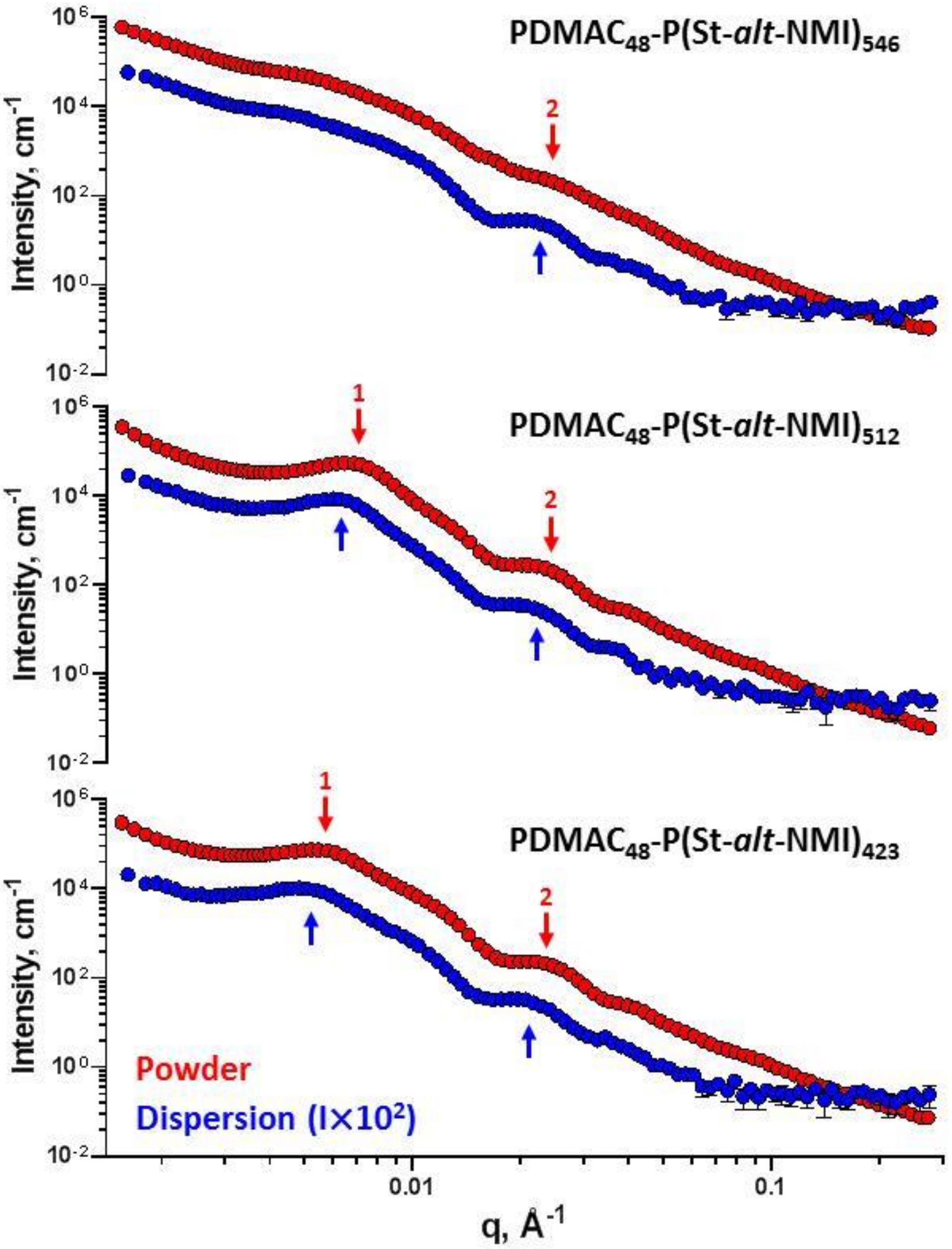

Copolymer composition		Peak Number 1		Peak Number 2	
		q (Å^{-1})	d (Å)	q (Å^{-1})	d (Å)
PDMAC$_{48}$-P(St-*alt*-NMI)$_{423}$	Powder	0.0058	1083	0.0234	269
	Dispersion	0.0051	1232	0.0206	305
PDMAC$_{48}$-P(St-*alt*-NMI)$_{512}$	Powder	0.0068	924	0.0221	284
	Dispersion	0.0063	997	0.0206	305
PDMAC$_{48}$-P(St-*alt*-NMI)$_{546}$	Powder	-	-	0.0247	255
	Dispersion	-	-	0.0229	274

Figure S8. SAXS patterns recorded for PDMAC$_{48}$-P(St-*alt*-NMI)$_{423}$, PDMAC$_{48}$-P(St-*alt*-NMI)$_{512}$ and PDMAC$_{48}$-P(St-*alt*-NMI)$_{546}$ in the form of either dried powders (red curves) or as 1.0% w/w dispersions in ethanol (blue curves). The data extracted from the SAXS patterns are summarized in the corresponding Table below.

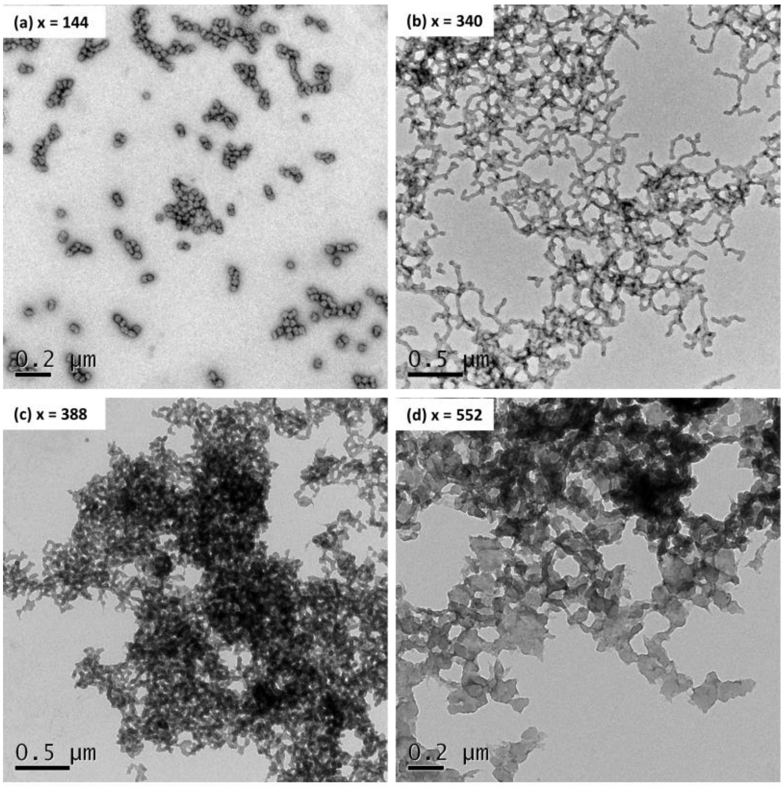

Figure S9. Representative TEM images illustrating the evolution in copolymer morphology for a series of $PDMAC_{48}$-P(St-*alt*-NMI)$_x$ diblock copolymer nano-objects prepared at 70 °C using a 50/50 w/w ethanol/**1,4-dioxane** mixture via RAFT alternating dispersion copolymerization of styrene with *N*-phenylmaleimide at 20% w/w solids: (a) spheres, (b) worms, (c) worm clusters, (d) ill-defined aggregates. Clearly, these copolymer morphologies differ markedly from those obtained when using the MEK co-solvent.

Table S1. Summary of the synthesis and characterization data obtained for a series of $PDMAC_{48}$–P(St-*alt*-NMI)$_x$ diblock copolymer nano-objects prepared via RAFT dispersion alternating copolymerization of styrene with *N*-phenylmaleimide at 70 °C using AIBN initiator in a 50/50 w/w ethanol/**1,4-dioxane** mixture at 20% w/w solids.[a]

Entry No.	Target DP for core-forming P(St-*alt*-NMI) block	Overall comonomer conversion[b] (%)	Actual DP for core-forming P(St-*alt*-NMI) block[c]	DMF GPC	
				M_n	M_w/M_n
**1**	150	96	144	29,300	1.35
**2**	350	97	340	52,600	1.65
**3**	400	97	388	55,100	1.64
**4**	600	92	552	71,900	1.69

[a] Conditions: [St]/[NMI] comonomer feed molar ratio = 1.0; [macro-CTA]/[AIBN] molar ratio = 10.
[b] Determined by ^1H NMR spectroscopy studies in d_6-DMSO.
[c] Actual DP for P(St-*alt*-NMI) block = target DP × overall comonomer conversion.

Table S2. Summary of five-point BET data obtained for the four $PDMAC_{48}$-P(St-*alt*-NMI)$_x$ diblock copolymers (x = 336-546) microparticles shown in **Figure S2**.

**$PDMAC_{48}$ -P(St-*alt*-NMI)$_{336}$**			
Relatively pressure P/P_0	Volume @ STP cc/g	1 / [W((Po/P) - 1)]	BET summary
0.095275	6.3250	13.321	Slope = 109.465<br>Intercept = 2.784<br>Correlation coefficient, r = 0.999870<br>C constant = 40.323<br>Surface Area = 31.025 m²/g
0.150022	7.3559	19.198	
0.195585	8.0903	24.046	
0.249745	8.8787	29.998	
0.296223	9.5183	35.381	
**$PDMAC_{48}$ - P(St-*alt*-NMI)$_{423}$**			
Relatively pressure P/P_0	Volume @ STP cc/g	1 / [W((Po/P) - 1)]	BET summary
0.106480	8.8195	10.811	Slope = 81.238<br>Intercept = 2.096<br>Correlation coefficient, r = 0.999886<br>C constant= 39.760<br>Surface Area = 41.790 m²/g
0.148551	9.8489	14.174	
0.197541	10.9154	18.045	
0.249785	11.9461	22.300	
0.295094	12.7934	26.181	
**$PDMAC_{48}$-P(St-*alt*-NMI)$_{512}$**			
Relatively pressure P/P_0	Volume @ STP cc/g	1 / [W((Po/P) - 1)]	BET summary
0.104476	11.1983	8.3356	Slope = 64.478<br>Intercept = 1.519<br>Correlation coefficient, r = 0.999822<br>C constant= 43.458<br>Surface Area = 52.768 m²/g
0.146675	12.5394	10.968	
0.196219	13.8770	14.075	
0.248542	15.1610	17.455	
0.300205	16.3546	20.987	
**$PDMAC_{48}$-P(St-*alt*-NMI)$_{546}$**			
Relatively pressure P/P_0	Volume @ STP cc/g	1 / [W((Po/P) - 1)]	BET summary
0.108325	10.4524	9.2994	Slope = 69.452<br>Intercept = 1.689<br>Correlation coefficient, r = 0.999839<br>C constant= 42.116<br>Surface Area = 48.952 m²/g
0.149778	11.6722	12.076	
0.198409	12.9013	15.351	
0.250073	14.0397	19.004	
0.295202	15.0326	22.293	

References

1. M. Semsarilar, E. R. Jones, A. Blanazs and S. P. Armes, *Adv. Mater.* 2012**, 24**, 3378-3382.

2. J. Ilavsky and P. R. Jemian, *J. Appl. Crystallogr.* 2009, ***42***, 347–353.
